# A Molecular Simulation Study of Silica/Polysulfone Mixed Matrix Membrane for Mixed Gas Separation

**DOI:** 10.3390/polym13132199

**Published:** 2021-07-01

**Authors:** Khadija Asif, Serene Sow Mun Lock, Syed Ali Ammar Taqvi, Norwahyu Jusoh, Chung Loong Yiin, Bridgid Lai Fui Chin, Adrian Chun Minh Loy

**Affiliations:** 1 CO_2_ Research Center (CO_2_ RES), Department of Chemical Engineering, Universiti Teknologi PETRONAS, Seri Iskandar 32610, Malaysia; khadija_20000006@utp.edu.my (K.A.); norwahyu.jusoh@utp.edu.my (N.J.); 2 Department of Chemical Engineering, NED University of Engineering and Technology, Karachi 75270, Pakistan; aliammar@neduet.edu.pk; 3 Neurocomputation Lab, National Centre of Artificial Intelligence, NED University of Engineering and Technology, Karachi 75270, Pakistan; 4 Department of Chemical Engineering and Energy Sustainability, Faculty of Engineering, Universiti Malaysia Sarawak (UNIMAS), Kota Samarahan 94300, Malaysia; clyiin@unimas.my; 5 Department of Chemical Engineering, Faculty of Engineering and Science, Sarawak Campus, Curtin University Malaysia, Miri 98009, Malaysia; bridgidchin@curtin.edu.my; 6 Department of Chemical Engineering, Monash University, Clayton, VIC 3800, Australia; adrian.loy@monash.edu

**Keywords:** CO_2_/CH_4_ gas transport, molecular simulation, empirical modelling, mixed matrix membrane, mixed gas, silica, polysulfone

## Abstract

Polysulfone-based mixed matrix membranes (MMMs) incorporated with silica nanoparticles are a new generation material under ongoing research and development for gas separation. However, the attributes of a better-performing MMM cannot be precisely studied under experimental conditions. Thus, it requires an atomistic scale study to elucidate the separation performance of silica/polysulfone MMMs. As most of the research work and empirical models for gas transport properties have been limited to pure gas, a computational framework for molecular simulation is required to study the mixed gas transport properties in silica/polysulfone MMMs to reflect real membrane separation. In this work, Monte Carlo (MC) and molecular dynamics (MD) simulations were employed to study the solubility and diffusivity of CO_2_/CH_4_ with varying gas concentrations (i.e., 30% CO_2_/CH_4_, 50% CO_2_/CH_4_, and 70% CO_2_/CH_4_) and silica content (i.e., 15–30 wt.%). The accuracy of the simulated structures was validated with published literature, followed by the study of the gas transport properties at 308.15 K and 1 atm. Simulation results concluded an increase in the free volume with an increasing weight percentage of silica. It was also found that pure gas consistently exhibited higher gas transport properties when compared to mixed gas conditions. The results also showed a competitive gas transport performance for mixed gases, which is more apparent when CO_2_ increases. In this context, an increment in the permeation was observed for mixed gas with increasing gas concentrations (i.e., 70% CO_2_/CH_4_ > 50% CO_2_/CH_4_ > 30% CO_2_/CH_4_). The diffusivity, solubility, and permeability of the mixed gases were consistently increasing until 25 wt.%, followed by a decrease for 30 wt.% of silica. An empirical model based on a parallel resistance approach was developed by incorporating mathematical formulations for solubility and permeability. The model results were compared with simulation results to quantify the effect of mixed gas transport, which showed an 18% and 15% percentage error for the permeability and solubility, respectively, in comparison to the simulation data. This study provides a basis for future understanding of MMMs using molecular simulations and modeling techniques for mixed gas conditions that demonstrate real membrane separation.

## 1. Introduction

The amount of carbon dioxide in the atmosphere has steadily increased after the beginnings of the industrial revolution (18th century) due to the rise in the burning of fossil fuels like coal, oil, and natural gas. The increase in the world temperature was about 0.74% in the most recent 100 years and is forecasted to reach 6.4% towards the end of the century [1]. Mainly found in natural gas streams, flue gases, and anaerobic digestion, CO_2_ has become a significant contributor to global warming [2]. During the processing of these fuel sources in the industrial sector, a reduction in the calorific value is observed, which subsequently reduces the possibilities of gas compression and transport within the transportation system due to the presence of CO_2_. The separation of CO_2_/CH_4_ has gained potential experimentation with many separation technologies to treat greenhouse gas emissions in the past decade [2]. These practical and economical techniques are being commercially utilized and analyzed to enhance the separation of CO_2_ from CH_4_ with a broad capture range of CO_2_ concentrations and transport properties [3,4].

There are many technologies formulated for the separation of CO_2_. The most used technologies are adsorption, membrane separation, absorption, biological separation, distillation, and separation by hydrates. Among these, the most popular and commonly used technology for CO_2_ capture is amine absorption. However, the energy required for solvent regeneration is a big challenge that reduces its economic feasibility [2]. Membrane separation has emerged as an economically friendly and effective alternative in the separation industry. Membrane technology has emerged from silicon rubber membranes to asymmetric and carbon membranes. It is an energy-efficient separation process that requires a detailed study of gas transport properties within the membrane material. Among inorganic, polymer, and hybrid membranes, the polymeric type is being used commercially to separate gases [5]. However, a tradeoff performance between permeability and selectivity has been observed. Deeming the importance of membrane technology and the requirement to overcome the challenges of separation performance posed by a commercial membrane, hybrid membranes, i.e., mixed matrix membranes (MMMs), are closely considered [4,6].

MMM is a popular field recently formed by incorporating fillers in the polymer matrix [7]. For dense polymer-based MMMs, the fillers used are inorganic material with distinguishing features (e.g., zeolites, silica, and carbon nanotubes) [8,9]. These features can be exemplified by selecting a suitable polymer that exhibits good compatibility with the filler. Polysulfone (PSF), for that matter, is a widely used glassy polymer membrane material that has been explored for gas separation. It exhibits good permeation properties for pure and mixed gas. PSF is also found to be an impartially attractive material for the incorporation of inorganic filler to enhance gas transport properties. In this context, PSF acts as the support system for gas particles to pass by, reaching the adsorption sites on the filler sites [10,11]. To elucidate better performing PSF-based MMMs, thermodynamics, and mechanics, the system has to be studied under varying conditions.

Nonetheless, preparation of PSF-based MMMs at the experimental scale is time-consuming, accompanied by a mismatch between inappropriate polymer base and inorganic filler, which contributes to increases in experimental cost with a requirement of multiple trials and errors [12]. In industry, the separation process in a membrane is observed under mixed gas conditions. Nonetheless, most experimental research works are studied under fixed feed and operating parameters under pure gas conditions, limiting the application in the actual separation industry. Moreover, transport properties like permeation and selectivity of gas molecules in pure pristine and filler-filled MMM need thorough conception at a molecular level, which is challenging to obtain under an experimental atmosphere [13,14,15,16]. Thus, the difficulties mentioned encumber the preparation and findings of a better-performing membrane. To understand the gas transport behavior for the real separation process, the empirical investigation of MMM under mixed gas conditions is an imperative addition that needs to be carried out with advertence in the quest for a suitable membrane for industrial application.

An alternative route to configure and analyze membranes is by applying computational chemistry to the membrane matrix. Molecular simulation of membranes has emerged as a mature technique to study the macromolecular structure of membranes. The simulation tools enable the instantaneous elucidation of material characteristics from an atomistic point of view, which circumvents the requirement of in situ measurements at varying operating conditions that are costly and not within the capacity limit of experimental instruments [17]. Molecular simulation provides a modeling and simulation environment designed to allow one to predict and understand the relationships of a material’s atomic and molecular structure with its properties and behavior, either by performing molecular dynamics (MD) or Monte Carlo (MC) simulations [18].

As computational chemistry is becoming an emerging tool for studying membrane and gas transport at the molecular level, many advanced simulation studies have been carried out. Erucar et al., 2012 made predictions on selectivity and permeability by performing atomistic simulations for H_2_/CH_4_ separation. The comparative study was based on MOF-based MMMs, which offered high H_2_ selectivity and permeability relative to the pure polymeric membrane [17]. Penetrant gas transport properties for industrial gases were studied by Golzar et al., 2014 by drawing a comparison among the pure and silica-filled PSF MMM. The simulations revealed consistency with the experimental results, depicting that the silica-filled PSF membrane possessed greater diffusion, solubility, and permeability coefficients [19]. However, the simulation result was confined to a pure gas study, which does not reflect a real system for gas separation. Hwang et al., 2018 supported his experimental work on ZIF-8@5FDA-DAM (ferrierite polyimide and zeolitic imidazolate framework) using MMM infrared microimaging with Grand Canonical Monte Carlo (GCMC) simulations, which elaborated the high concentration interfacial layer of CO_2_ molecules over MOF filler in the microvoids. The reported research helps to understand the preferential location of CO_2_ in this MOF/polymer combination [20]. Liu et al., 2019 adopted a graphene-like filler, i.e., molybdenum disulfide (MoS_2_), integrated into PEBAX-1657 (60 wt.% polyethylene oxide (PEO) and 40 wt.% polyamide (PA)) to study CO_2_/N_2_ solubility and selectivity. The molecular simulations were performed using the Metropolis MC method, which displayed significant improvement by incorporating MoS_2_ nanosheets in PEBAX membranes with increments in CO_2_/N_2_ solubility selectivity. The results concluded that the MoS_2_ nanosheet is a competent filler to be considered for CO_2_ separations in MMMs [21]. Recently, a fair number of studies have been conducted on polysulfone (PSF) using molecular simulation considering its vast application to understand its morphology and behavior under different atomic forces, including interfacial properties and cavity size distribution [11,16,22,23,24,25]. A soft confining methodology was employed by Lock et al., 2019, to study the physical properties (i.e., glass transition temperature, density, cavity sizes) of ultrathin PSF polymeric membrane, which resulted in a better understanding of an atomistic-level using molecular simulation [26]. Although an increasing number of simulation works using PSF polymeric membrane have been carried out to elucidate gas separation performance, the number of works is still limited compared to a myriad of experimental results available.

As the addition of a suitable filler can enhance the performance of a membrane, PSF has been paired with different inorganic fillers. Among the available fillers, silica has been found to have eminent properties that have contributed to enhancing membrane transport properties [14,27,28]. The addition of silica has improved gas permeability and selectivity with variation in the size or shape of the fillers [29,30]. In this context, the dispersion of properly designed silica in the membrane matrix enhances the diffusion ability of larger gas molecules by repositioning the polymer chain packing order [31]. Previous studies showed that the permeability of the single gases (i.e., H_2_, N_2_, O_2_, CO_2_, CH_4_) increased with the incorporation of silica [32,33]. The results of single gas conditions also concluded that the permeability and selectivity could be regulated by altering the type and concentration of silica in PSF-based MMMs [27]. The major elucidations of PSF-based MMMs have usually been conducted using atomistic simulation under a single gas condition with changing temperature, pressure, and filler loadings to enhance gas transport performance. However, it should not be overlooked that transport characteristics in real systems and separation are achieved by separating mixed gases. To explain the effect of varying weight percentages of silica on gas transport properties, this can be achieved by studying the interaction of molecules in a mixed gas system for silica/PSF-based MMM. Although the effect of silica concentration has been proven to enhance permeability and diffusion coefficient of the membrane compared to its pristine polymer, the study under mixed gas conditions that resembles real gas separation is yet to be resolved. Therefore, the novelty of this work is to elucidate the effect of mixed gas with varying CO_2_/CH_4_ concentrations towards gas transport behavior of silica/PSF-based MMMs at different filler loadings using molecular simulation tools and to develop an empirical model to quantify the effect of gas concentrations in MMMs.

In this research, molecular simulation was kept as the basis of the study to understand the morphological, physical, and gas transport properties of silica/PSF-based MMM. Temperature and pressure were kept constant to keep the focus of the study on the effect of CO_2_ and CH_4_ gas transport properties under single and mixed gas conditions. The silica/PSF-based MMM behavior under changing mixed gas concentrations of CO_2_/CH_4_ (i.e., 30%, 50%, 70%) and silica loadings at different weight percentages (i.e., 15, 20, 25, 30 wt.%) were adhered to consistently in the simulation work. The different mixed gas concentrations are essential to reflect the actual natural gas sweetening process with varying CO_2_/CH_4_ contents and to study the influence that one gas exerts on the permeation of the other gas molecules [34,35]. The simulation results are later quantified by developing an empirical model to study gas concentrations in silica/PSF-based MMMs. This work demonstrates the advantages of molecular simulation to determine gas transport properties of sufficient accuracy in a less time-consuming and cost-effective manner, which can be used to screen, evaluate, and develop an appropriate empirical model to describe the separation performance of membranes [36].

## 2. Methodology

The molecular simulation methodology for this study is reported in three sections. Section 2.1 emphasizes the construction of the reference PSF chain and the silica structure for the polymeric membrane. The free volume distribution in the membrane matrix and the X-ray diffraction pattern are discussed in Section 2.2 and Section 2.3. The discussion on gas transport properties (i.e., solubility, diffusivity, permeability) and the development of the empirical model is subsequently present in Section 2.4 and Section 2.5. The process is being carried out using Dassault Systèmes BIOVIA Materials Studio (Version 8, San Diego, CA, USA) software to formulate the structures and validate the process parameters [37]. The operating modules and specifications for the process are shown in Table 1. Since interaction among molecules must adhere to quantum mechanics descriptions, a suitable force field must ensure an accurate record of these interaction forces. In this work, the interactions among the gas molecules, polymeric material, and filler were studied using the Condensed Phase Optimized Molecular Potentials for Atomistic Simulation Studies (COMPASS) force field. The COMPASS forcefield was used consistently for single and mixed gas conditions. It helps reduce the time of calculation by only evaluating strong interactions and is suitable for a polymeric system [38]. The COMPASS force field also enables the simulated cell to fall near the PSF final structural density range, which is consistent with reported experimental values [29].

### 2.1. Simulation of Molecular Structures

In this section, the construction of the silica and PSF, which was used for the simulation of a membrane system in this work, is discussed in detail. The silica used in the simulation is α-quartz crystal silica with a 6.1 Å radius constructed using the “Build” module of the software. The nanocluster used had 26 Si and 52 O atoms. The excess atoms above the stable occurrence state were removed to coincide with the properties reported in the literature from experimental observation [27].

**Figure 1:**
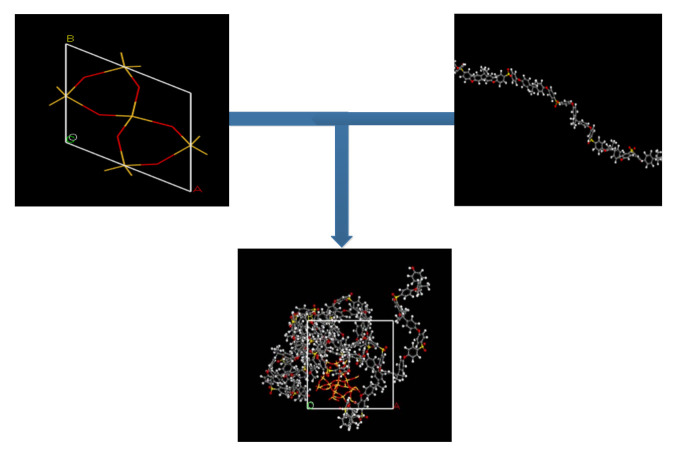
Illustration of incorporation of silica nanocluster in polysulfone chain to form silica/PSF MMM.

To maintain a structural construction within periodic boundary conditions (PBC), it is encouraged to avoid the finite size effect through the employment of a polymer with the same chain length throughout the cell [27,39]. To ensure an effective physical system, a shorter chain is preferred, as it enhances the mobility of the polymeric chain regardless of the cell dimensions [40,41]. A PSF chain with 20 repeat units was used consistently for simulating all polymeric membranes. The PSF chain was constructed according to steps reported by Golzar et al., 2014, since it has been demonstrated to be successful in simulating PSF membrane with good compliance with experimental observation [26,29,42]. The silica nanocluster was later incorporated within the PSF polymer with varying silica weight percentages to study the behavior of mixed gas transport through the MMM. The incorporation of the α-quartz silica nanocluster into PSF is displayed in Figure 1 with the following atom representation: white, hydrogen; grey, carbon; yellow, sulfur; red, oxygen; light yellow, silicon.

For silica/PSF-based MMM with varying silica weight percentages (i.e., 15, 20, 25, 30 wt.%), they were built by incorporating the silica nanocluster with the polysulfone polymer chain. In this context, the silica and PSF were incorporated within the same amorphous cell simultaneously with varying pristine material to comply with the weight percentages. Details of PSF chains, silica nanoparticles, and cell lengths of the simulated membranes are displayed in Table 2.

The initial density of the simulated cell was set at 1.20 g/cm^3^ under medium quality, which corresponds to the density of PSF (i.e., 1.24 g/cm^3^) measured from experimental work. The temperature was set to 308.15 K at 1 atm. The silica/PSF structure was pleated under periodic boundary conditions in the amorphous cell. To eliminate overlapping and adjacent contact, 10,000 energy minimization steps were adopted. The annealing process was conducted by performing iterative NPT MD using a temperature cycle protocol between 308.15 and 508.15 K at an interval of 25 °C. The heating protocol was performed up to 508.15 K, which is above the glass transition temperature (T_g_) of the PSF polymer [43]. The annealing strategy was applied to the membranes structures as it withstands high-temperature treatment during each cycle to find different local minimum energy structures while eliminating the residual internal energy before being subjected to the MD protocol to overcome energy barriers. The MD was intended to achieve the ideal structure at the lowest energy confirmation with a dimensional change in the silica/PSF MMM cell structure.

To achieve the ideal structure with the lowest energy conformation and to obtain an equilibrium density to be compared with experimentally observed values, an additional 500 ps NPT MD simulation course was executed on the system using the “Forcite” module. The number of molecules, *N*, the pressure, *P*, and the temperature, *T*, were kept constant during the MD procedure. A Nose–Hoover–Langevin (NHL) thermostat and an Andersen barostat were adopted to regulate the pressure at 1 atm and temperature at 308.15 K. The summation method used to calculate the electrostatic interactions was supported by the Ewald method with an accuracy of 0.001 kcal/mol, and the van der Waals interaction was observed under atom-based selections with a cut-off distance of 12.5 Å, which was integrated at a time-step of 1 fs [29].

### 2.2. Fractional Free Volume

Morphology and free volume are determining factors of molecular diffusion through a polymer membrane [11]. Static voids are created by chain packing or transient gaps produced by thermally induced chain rearrangement. They add up to create the free volume, which provides the diffusing molecules with a low resistance path for their mobility [44,45]. The membrane configuration and the channel of the gas transport are better understood by the presence of void space, which is studied through the free volume concept. There are two phases: one consists of a solid phase comprising all the polymeric chains, and the other with a space region called the free volume. The portion of the void region within the membrane is also known as fractional free volume (FFV) [27,29], as displayed in Equation (1),

(1) $$FFV=\frac{v_{g}-v_{o}}{v_{g}}$$

where the specific volume of the polymer is described by *v_g_*, while the occupied volume is denoted as *v_o_*. The free volume calculated in this work followed a medium grid resolution for all the amorphous cells with changing weight percentages of silica throughout the simulations. The Connolly surface was studied with 0.4 Å grid intervals to observe the void space that acts as a pathway for gases to pass through the membrane matrix.

### 2.3. X-ray Diffraction Pattern

The X-ray diffraction (XRD) of all simulated membrane structures can be independently calculated under the equilibrium arrangement of Cu rays, *λ* =1.54 Å, in the periodic boundary state using the Forcite scattering analysis module. The scattering intensity following a Fourier transform operation is represented in Equation (2),

(2) $$I\left(Q\right)=\underset{j}{\sum }\underset{k}{\sum }b_{j}b_{k}\left(sinQr_{jk}\right)/\left(Qr_{jk}\right)$$

where the indices *j* = *k* imply that summations are applied on all atoms in the membrane system, and *r* is the distance between atoms *j* and *k* with *b* as the atomic scattering coefficient. The magnitude of the scattering angle can be calculated using Equation (3),

(3) $$Q=\frac{4\pi sin\theta }{\lambda }$$

where *Q* is the magnitude scattering vector, *λ* is the radiation wavelength, and *2θ* is the scattering angle [46]. The highest peak of the diffraction pattern is very significant as it contributes to calculating the d-spacing, which represents the intersegmental distance between the polymer chains. The highest peak ascribed to d-spacing was calculated using Braggs’s law following real-time coupling using Equation (4) [47].

(4) $$d=\lambda /\left(2sin\theta \right)$$

### 2.4. Gas Transport Properties Study

#### 2.4.1. Solubility

The solubility of the silica/PSF membrane was calculated by adopting the adsorption isotherm in the “Sorption” module. COMPASS forcefield was used to calculate the non-bonded energy of all the molecules in the membrane matrix. The sorbate molecules (i.e., CO_2_, CH_4_) were incorporated in the cells and observed under PBC for pure and mixed gas conditions. Each set of amorphous cells was dealt with as a rigid body applying the Metropolis method to give smaller sorbate molecules a path to pass through the pores. The selected structure from the end of the 500 ps NPT MD step, which had reached an equilibrium state, was used to proceed with the solubility study. The final solubility was achieved by observing the resulting isotherm, whereby it could be determined from the slope of the concentration versus pressure curve at zero pressure limit. The solubility was calculated as 308.15 K at 1 atm, which was later compared to the reported experimental values in reference [29]. The solubility coefficient of gas species *i*, $S_{i}$, was calculated using Equation (5) [32].

(5) $$S_{i}=\underset{p\to 0}{lim}\left(\frac{C_{i}}{P}\right),$$

with *P* as pressure and *C_i_* as the concentration of species *i* in the polymer with the unit in cm^3^(STP)/cm^3^, where STP is standard temperature and pressure. The same process was repeated to calculate the solubility of CO_2_ and CH_4_ under mixed gas conditions. However, for mixed gas simulation, the adsorption isotherm was calculated until the partial pressure of the gas instead of total pressure as observed for its pure counterpart.

#### 2.4.2. Diffusivity

The diffusivity of the silica/PSF-based MMM was calculated by subjecting the gas molecules to be part of the membrane cell during construction using the Amorphous Cell module. In this context, both CO_2_ and CH_4_ were added separately for pure gases and together with different concentrations for the mixed gas calculations. For mixed gases, the gas molecules were added with different concentrations (i.e., 30% CO_2_/70 %CH_4_, 50% CO_2_/50% CH_4_, 70% CO_2_/30% CH_4_) to observe the diffusivity path of the molecules in close relevance to a real gas separation system. The silica/PSF-based MMM with the presence of gas molecules was geometrically optimized and energy minimized before undergoing 500 ps NPT. The NPT treatment was to allow dispersion of gas molecules within the membrane matrix. This set of amorphous cells with varying silica weight percentages and CO_2_/CH_4_ concentrations were relaxed after reaching an equilibrium state at 308.15 K at 1 atm with gas molecules. The gas molecules were named in sets to easily trace the pathway for each molecule passing through the membrane matrix. Later, a Canonical ensemble (constant number of molecules, volume, and temperature) or NVT simulation was performed for 5000 ps to study the diffusion of the gas molecules in the three-dimensional membrane matrix. The thermostat used was a Nose-Hoover-Langevin (NHL), which is a popular thermostat used to control the temperature of a simulation. It was maintained with a Q ratio of 0.005 and a decay constant at 0.1 ps. In addition, NHL is considered a reliable thermostat to be used together with an Andersen barostat (with a cell time constant of 0.5 ps) for a system that has achieved an equilibrium state [18]. The diffusivity coefficients of the gas species *i*, $D_{i}$, are calculated by Einstein Equation (6) [32].

(6) $$D_{i}=\frac{1}{6N}\underset{n\to \infty }{lim}\frac{d}{dt}\overset{n}{\underset{t=1}{\sum }}{\left|r_{i}\left(t\right)-r_{i}\left(0\right)\right|}^{2}>,$$

The equation was used to determine the average mean square displacement (MSD) of the gas molecule trajectories $\left|r_{i}\left(t\right)-r_{i}\left(0\right)\right|$^2^, where *r_i_* is the position vector of the atom *i*, and *N* is the number of diffusing atoms. The diffusion coefficients were calculated at normal diffusion regime right after each set achieved a slope change approaching unity for the CO_2_/CH_4_ concentrations. The diffusion coefficient is calculated from the normal diffusion regime based on the exponential parameter, *n*, shown in Equation (7).

(7) $$<|r_{i}{\left(t\right)}^{2}\;-\;r_{i}{\left(0\right)}^{2}|>\propto r^{n}$$.

In the anomalous regime, *n* is less than 1, which approaches 1 at the onset of normal diffusion at sufficiently long simulation time, in which the transport property can be quantified via the Fickian behavior, as reported in various literary works [32,47].

#### 2.4.3. Permeability and Selectivity

Diffusivity and solubility are the two affecting attributes of permeability and consistently respond with any changes in the transport properties [27,48]. The permeability of the gas species *i* was calculated by Equation (8), which was adopted in the reported literature to study the behavior of the gases passing through the membrane matrix [48,49].

(8) $$P_{i}=D_{i}S_{i},$$

where $D_{i}$ and $S_{i}$ are the coefficients of diffusivity and solubility, respectively, for gas *i* passing through the membrane matrix. The selectivity of a membrane can be found by the calculation ratio of the permeability of the gases passing through the membrane. The ideal gas selectivity of a membrane can be calculated by Equation (9),

*α_ij_* = *P_i_/P_j_*, (9)

where *α_ij_* is defined as the ratio of the permeability of two pure gases measured separately under the same conditions. For mixed gas conditions, perm-selectivity or true selectivity can be calculated by the ratio of the permeability of mixed gases passing through the membrane matrix. It is important to study the true selectivity, as it can deviate substantially from ideal selectivity due to interaction or competitive adsorption–diffusion between two gases [34,50].

### 2.5. Empirical Modeling

It has been reported by Ahn et al., 2008 [29] that the Maxwell model is not able to predict the effect of silica nanoparticles in PSF-based MMM for pure gas separation, which is consistent with the published literature, that the suitability of the Maxwell model on prediction of MMM performance is dependent upon the inorganic filler, polymer type, and loadings [51,52,53]. In addition, most of the studies employing the Maxwell model were confined to pure gas permeation, while its applicability for mixed gas separation has received less scrutiny to date [54,55,56]. Hence, to address the limitation of the Maxwell model in describing transport behavior of silica/PSF based MMM and further extending it to incorporate mixed gas concentrations, we employed a simple parallel resistance model in the present study to be consistent with the density evolution in PSF MMM with the presence of silica, as reported by Ahn et al. (2008). In addition to being simple in implementation, the model can circumvent the contradictory prediction by the Maxwell model, as reported in work of Ahn et al., 2008 while incorporating the effect of mixed gas concentrations and filler loadings towards gas transport behavior in PSF/silica MMM [29]. An empirical model was developed for a heterogeneous medium, and the simulation results were later compared with the model results. The concepts of permeability and selectivity were introduced to integrate their mathematical formulation into the model to study the transport in the MMMs. The model was developed following the resistance model approach (RMA) in which effective permeability is usually based on permeabilities and volume fractions of the MMMs [57,58]. However, in the developed parallel model, the effective permeability was calculated using the permeability and weight fractions of the silica/PSF-based MMM constituent phases. The model calculations were kept consistent with the simulation operating parameters. The model can be applied to incorporate the effect of filler morphology (e.g., size, shape, and agglomeration), polymeric base, and mixed gas concentrations with varying gas species. At moderate pressure (i.e., 1 atm) and varying independent variables, the solubility of gas species *i* in the filler, $S_{fi}$, was calculated using Equation (10), which explains the sorption equilibrium well in silica [59].

(10) $$S_{fi}=K_{fi}C_{fi}^{s}/\left(1+\sum_{i=1}^{N}x_{i}K_{fi}f\right),$$

where $K_{fi}$ is the affinity constant, $C_{fi}^{s}$ is the capacity in the filler, and f is the total fugacity of the gas species *i* with mole fraction $x_{i}$.

The diffusivity was assumed to be concentration-independent and contributed to the calculation of gas permeability for the filler phase, $P_{fi}$, using Equation (11),

(11) $$P_{fi}=D_{fi}K_{fi}C_{fi}^{s}/\left(1+\sum_{i=1}^{N}x_{i}K_{fi}f\right),$$

where *D_fi_* is the permeant diffusivity of the gas species *i* in the filler. A combination of Henry’s law and the Langmuir model was used to calculate the polymer solubility, $S_{ci}$, following Equation (12),

(12) $$S_{ci}=K_{hi}+K_{ci}C_{ci}^{s}/\left(1+\sum_{i=1}^{N}x_{i}K_{ci}f\right),$$

where $K_{hi}$ is Henry’s constant, $K_{ci}$ the affinity constant, and $C_{ci}^{s}$ the capacity in the polymer membrane base. The permeability of the polymer, $P_{ci}$, was thus calculated using Equation (13),

(13) $$P_{ci}=D_{hi}K_{hi}+D_{ci}K_{ci}C_{si}^{c}/\left(1+\sum_{i=1}^{N}x_{i}K_{ci}f\right)$$

where $D_{hi}$ and $D_{ci}$ are the diffusivities in the Henry’s and Langmuir sites, respectively. These equations were applied to calculate the effective permeation, $P_{eff,i}$, and solubility, $S_{eff,i}$, with different gas concentrations of gas species, *i*, for the parallel resistance model using the final Equations (14) and (15),

(14) $$P_{eff,i}=P_{fi}\varphi +P_{ci}\left(1-\varphi \right),$$

(15) $$S_{eff,i}=S_{fi}\varphi +S_{ci}\left(1-\varphi \right),$$

where φ is the weight percentage of the filler used in the silica/PSF-based MMM. From Equation (10) to Equation (15), it is seen that the model considers the effect of species *i* with mole fraction $x_{i}$ and filler loading φ, for real gas separation in silica/PSF MMM.

## 3. Results and Discussion

### 3.1. Physical Properties

#### 3.1.1. Amorphous Cell Equilibrium

**Figure 2:**
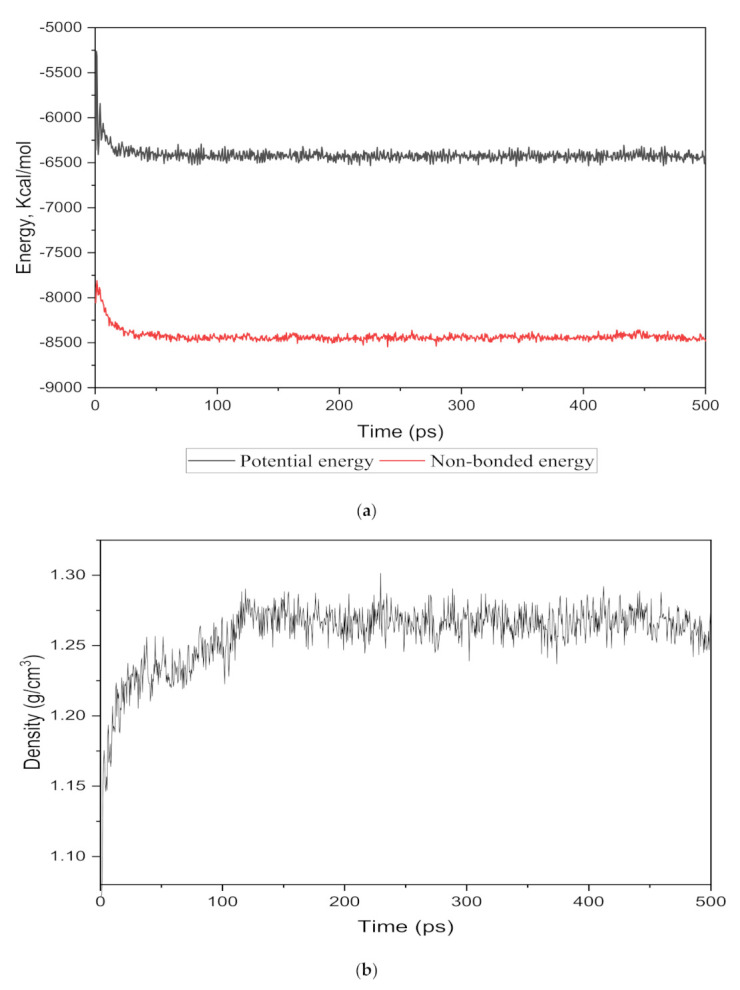
The (**a**) energy and (**b**) density evolutions during 500 ps NPT molecular dynamics simulation for 15 wt.% silica/PSF MMM.

The energy evolution and simulation density were observed as an equilibrium parameter for each cell structure to compare the nanocomposite membranes with the real membranes. The total potential and non-bonded energies vs. time step for the 15 wt.% silica as an example are depicted in Figure 2a. It was observed that the total energy decreased from −5500.046 kcal/mol to −4035.282 kcal/mol and after 350 time steps became approximately fixed at −4091.75 kcal/mol with few fluctuations. From the total energy with reduced deviations, it could be concluded that the system in the 500 ps time-step reached a thermodynamic equilibrium state.

To monitor the equilibrium state for the amorphous cell, an exemplary density result for 15 wt.% is shown in Figure 2b. It was observed that after approximately 120 time steps, the density curve was roughly fixed at 1.26 g/cm^3^, which was in close agreement with the experimental value reported in reference [29,32].

Similarly, Table 3 shows the corresponding densities achieved for different weight percentages of silica (15–30 wt.%) after maintaining an equilibrium between 100 and 150 time steps. The densities of the simulated cells were found to be increasing with an increasing weight percentage of silica. This effect was observed due to the incorporation of increasing wt.% of the filler, which has a higher density than the PSF polymer. No significant differences in the densities were observed after the addition of the gases under pure and mixed conditions. The minor difference between simulated and experimental observed values verified that the simulation procedure was accurate enough to proceed with the gas transport study through the silica/PSF-based MMM [29].

#### 3.1.2. Free Volume Characteristics

**Figure 3:**
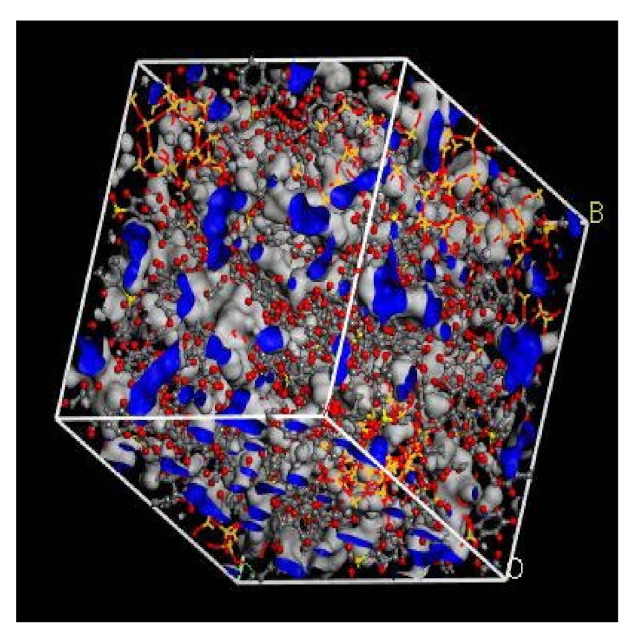
Illustration of fractional free volume with silica for 30 wt.% within PSF-based MMM.

**Figure 4:**
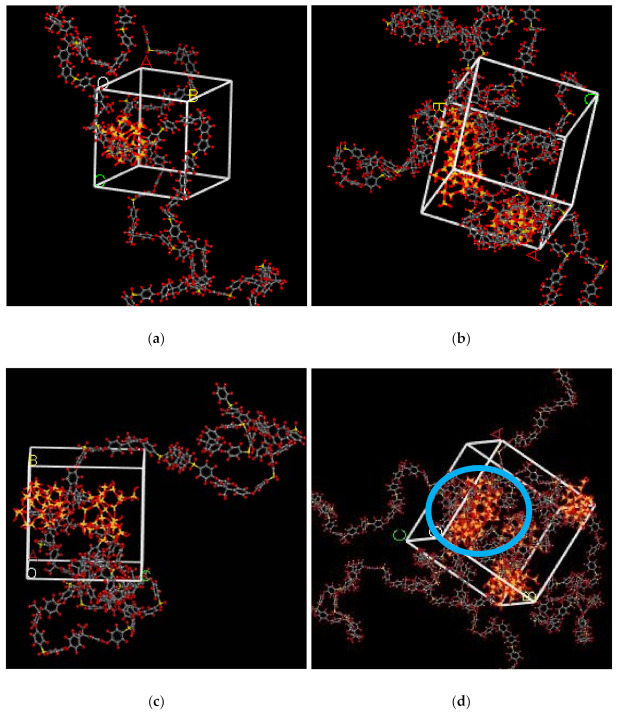
Illustration of amorphous cells for (**a**) 15 wt.%, (**b**) 20 wt.%, (**c**) 25 wt.%, and (**d**) 30 wt.% silica/PSF MMM after NPT with observed agglomeration marked by blue circle ring.

In the simulation, the increase in the silica particles disrupted the chain packing, which created more free volume. The increase was observed due to the less efficient packing of the polymer chain with increasing weight percentages of silica, which created voids at the interface between the polymer and the silica nanocomposites. The fractional free volume in the amorphous cells was found to be increasing with increasing wt.% of silica (i.e., for 15 wt.% 0.183, 20 wt.% 0.223, 25 wt.% 0.257, 30 wt.% 0.281) for mixed gases. The highest free volume was observed for 30 wt.% of the silica particles, as displayed in Figure 3, where grey color represents the occupied volume, and blue color depicts the free volume. In particular, for 30 wt.% silica/PSF MMMs, the increment in free volume was attributed to the stronger interaction between the inorganic filler of high weight percentage. The proximity of silica nanoparticles with one another caused the creation of bigger voids due to the agglomeration, as shown in Figure 4d.

#### 3.1.3. X-ray Diffraction (XRD)

**Figure 5:**
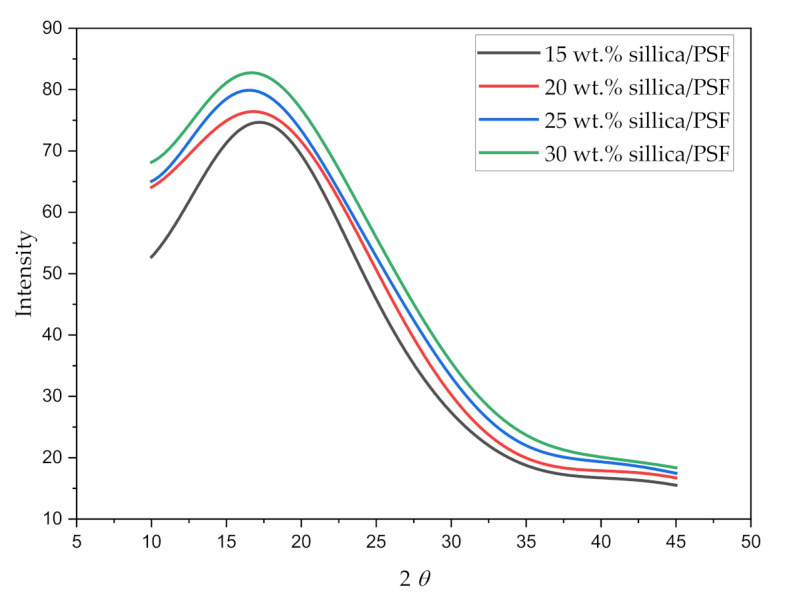
X-ray scattering pattern of varying silica weight percentages in silica/PSF MMMs.

The X-ray diffraction pattern for all the simulated membrane structures is displayed in Figure 5. The values of maximum intensity of the polymer (2θ) in the amorphous state and the d-spacing of the silica/PSF MMMs are reported in Table 4.

The wavelength 2θ when varied from 10° to 45° displayed a sharp peak for each silica/PSF MMM, which depicts the crystalline region of the membrane with the main peak of each sample located between 2θ≈16° and 18°. It can be concluded from the higher peaks that the addition of silica in the PSF indicates higher crystallinity in the MMMs [60,61]. The d-spacing of the simulated silica/PSF MMMs was found to be consistent when compared with the reported simulation XRD from the published literature [19,62]. Its d-spacing also revealed that the increasing silica from 15 to 25 wt.% in PSF extended the distance between the PSF chains due to disruption of fillers, which was consistent with the observation of increment in FFV. At 30 wt.% silica, the d-spacing decreased due to the agglomeration of silica at high crystallinity that reduced the distance.

### 3.2. Gas Transport Properties

#### 3.2.1. Gas Solubility (CO_2_/CH_4_)

**Figure 6:**
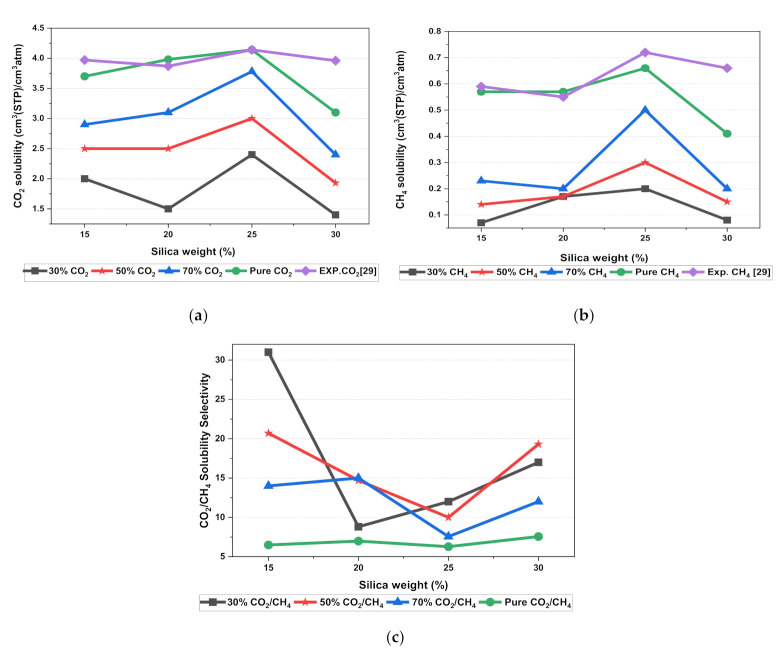
Solubility graphs for (**a**) CO_2_, (**b**) CH_4_, and (**c**) CO_2_/CH_4_ selectivity.

In this section, the position, orientation, and amount of the gas molecules were tested to study the sorption behavior of the silica/PSF-based MMM. The illustration of the amorphous cell structures used to perform sorption for different weight percentages of silica is displayed in Figure 4a–d. Figure 6 displays the evaluation of the solubility coefficients, depicting that the increasing gas concentrations followed an increase in the solubility value of carbon dioxide as 70% CO_2_ > 50% CO_2_ > 30% CO_2_, and similarly for methane, i.e., 70% CH_4_ > 50% CH_4_ > 30% CH_4_. It also shows that all the pure gas sorption values were greater than those calculated for mixed gases, whereas, the calculated solubility values for pure gas were in close agreement with the solubility values reported by Ahn et al. [29].

When compared to the pure silica/polysulfone, the solubility values for mixed gases showed a consistent decrement regardless of the weight percentage of the nanocomposite particles. This pattern was observed due to the competitive transport behavior of gases in the presence of mixed gas molecules. This is apparent when the percentage of one specific gas is increased, which subsequently suppresses the transport property of other mixed gases in the matrix [63,64]. CH_4_ molecules lack torsional flexibility in the case of mixed gas as the dominant gas. In this context, CO_2_ suppresses the pathway of the submissive CH_4_ gas passing through the voids that are created in the membrane due to filler incorporation [31,39]. This proves that the competitive nature of the gas molecules does contribute to further decrement in the solubility of the mixed gas. The solubility values for mixed gases increased with their increasing gas concentration due to the dominancy effect during competitive sorption in the membrane matrix. All mixed gas simulation results showed consistently higher solubility values for CO_2_ as compared to CH_4_, since CO_2_ has a higher critical temperature (i.e., 31.1 °C) in comparison to CH_4_ (i.e., −81.9 °C).

In addition, the results depicted an increase in the solubility coefficient with an increase in the silica nanoparticles up to a certain weight percentage and then dropped when more filler was further added. In this simulation, the solubility value showed an increment up to 25 wt.% silica and then a decrease in the solubility for 30 wt.% silica. The increase up to 25 wt.% by exhibiting the highest sorption value was due to the increase in the free volume of the polymer in the filled membrane. The same solubility pattern was observed in the study reported by Ahn et al. for silica/PSF MMM [29].

As silica has been eminent in providing the interfacial interaction among the gas molecules, the particle distribution in the membrane matrix is crucial to determine the gas transport properties under mixed gas conditions. When the gas molecules pass through the membrane matrix, the silica particles are expected to stay well dispersed without any aggregation to rearrange the chain packing of the polymer. It was seen in this simulation study that silica particles stayed well dispersed up to 25 wt.% silica [48,49,65,66,67]. The presence of silica increased the solubility of the membrane consistently for pure and mixed gases under such conditions. However, as the weight percentage of the silica was further increased up to 30 wt.%, it caused agglomeration, as observed in Figure 4d. This was due to the presence of higher intermolecular forces among the silica molecules. A decrease in the solubility values was observed for 30 wt.% silica/PSF MMM. This occurred due to close contact between the silica molecules, which acted as a barrier for the smaller molecules to pass through them, although larger cavity sizes were created during agglomeration. 

It was also seen that the CO_2_/CH_4_ selectivity for mixed gas concentrations showed a decrement, while selectivity for the pure gas condition increased with an increasing weight percentage of silica. As the free volume increased with an increasing weight percentage of silica, it allowed passage of both CO_2_ and CH_4_, which caused a decrement in the solubility selectivity of CO_2_/CH_4_ in silica/PSF-based MMM.

**Figure 7:**
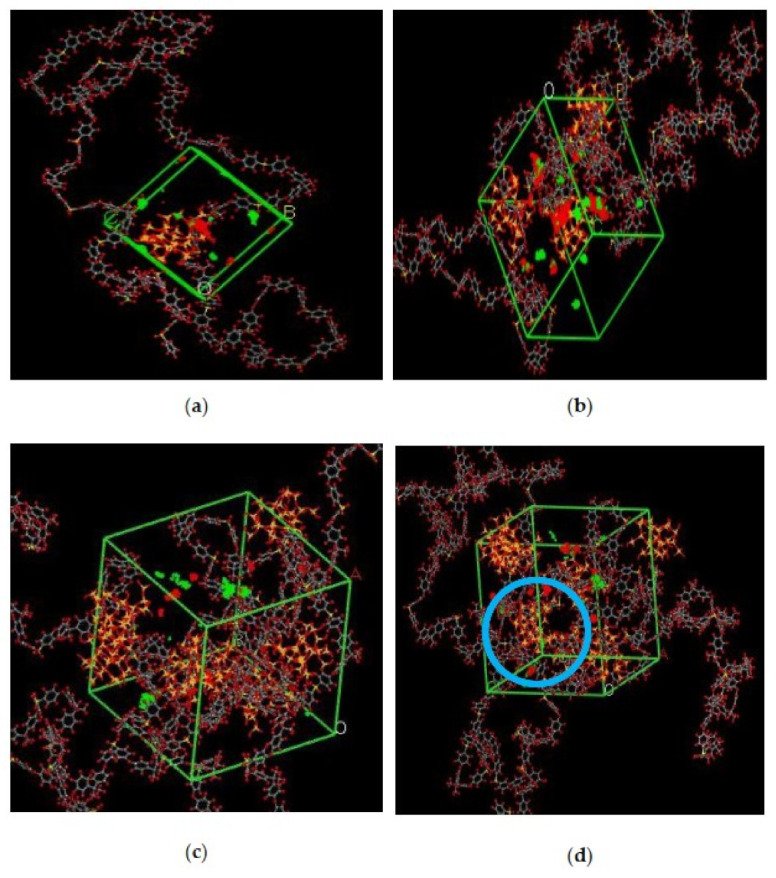
Active sorption sites of CO_2_/CH_4_ for silica/PSF MMM with (**a**) 15 wt.%, (**b**) 20 wt.%, (**c**) 25 wt.%, and (**d**) 30 wt.% inorganic filler at 50%CO_2_/50%CH_4_ with observed proximity marked by a blue circle ring.

**Figure 8:**
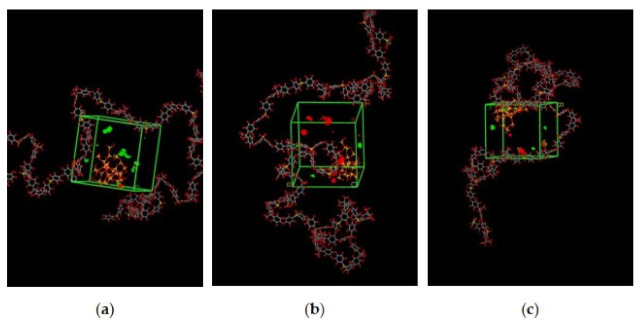
Active sorption sites of CO_2_/CH_4_ for silica/PSF MMM with (**a**) 30%CO_2_/70%CH_4_, (**b**) 50%CO_2_/50%CH_4_, and (**c**) 70%CO_2_/30%CH_4_ at 15 wt.% inorganic filler.

The illustration of active sorption sites for mixed gases with increasing weight percentages is displayed in Figure 7. The red and green colors display the active sorption sites for CO_2_ and CH_4_ as the gases with different concentrations passed through the membrane matrix. As observed in Figure 7, the active sites for CO_2_/CH_4_ were found to be overlapping due to similar sorption sites involved in the process of absorption for the membrane. A consistent increase in the concentration of the gases in the sorption sites was observed with an increasing gas concentration up to 25 wt.%. However, fewer active sites were observed for 30 wt.% of silica, as the solubility of the gases decreased due to agglomeration observed in Figure 7d, which caused difficulty in sorption of the gas molecules. The solubility values were found to be increasing with increasing gas concentrations, as shown in Figure 8 for 15 wt.% of silica as an example with prominent active sorption sites.

**Figure 9:**
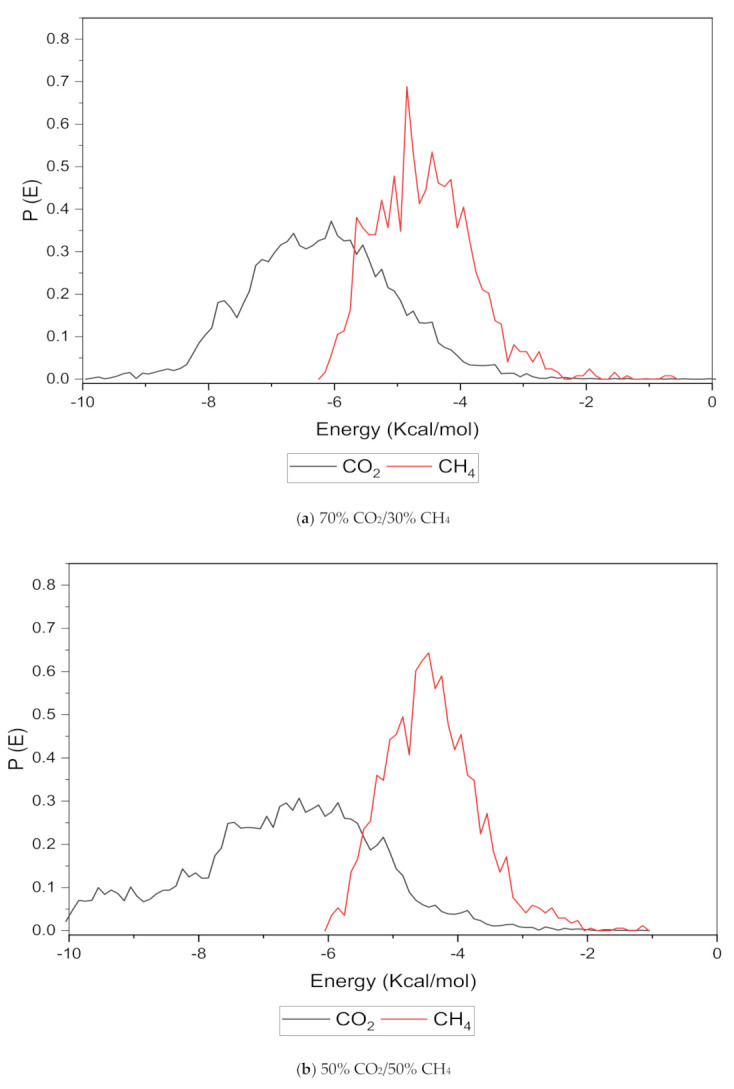

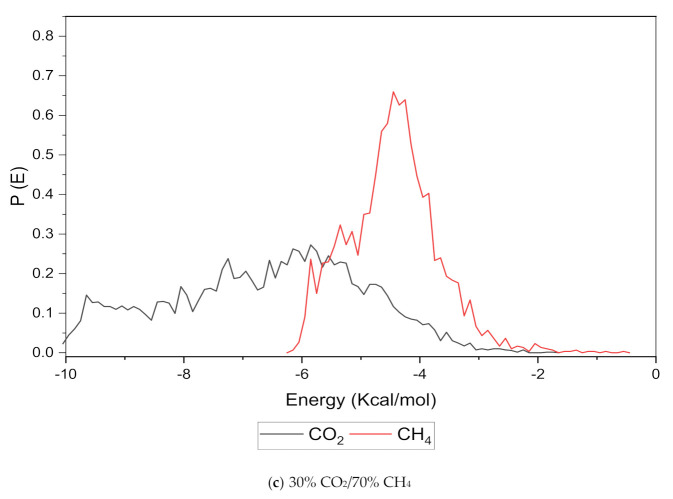
Effect of 15 wt.% of silica towards sorption energy distribution with gas concentrations for silica/PSF MMM. (**a**) 70% CO_2_/30% CH_4_; (**b**) 50% CO_2_/50% CH_4_; (**c**) 30% CO_2_/70% CH_4_.

An increase in the sorption capacity can be supported by higher and broader peaks observed for the energy distribution, which measures the interaction between the CO_2_ and CH_4_ sorbates with the membrane material [31]. For all weight percentages of silica, the solubility of CO_2_ was more pronounced than CH_4_ through broader energy distribution. An example of this consistent broader energy distribution for CO_2_ than CH_4_ is displayed for 15 wt.% in Figure 9. It was also observed that CO_2_ demonstrated a broader energy spectrum at 70% concentrations, as depicted in Figure 9a, as compared to its counterpart with lower gas concentrations in Figure 9b,c. For CH_4_ sorption, it was demonstrated to exhibit higher energy peaks when its concentration increased to 70%, as seen in Figure 9c, which reflected the higher affinity with membrane material when the presence of CH_4_ gas molecules was dominant due to less competitive transport.

#### 3.2.2. Gas Diffusivity (CO_2_/CH_4_)

**Figure 10:**
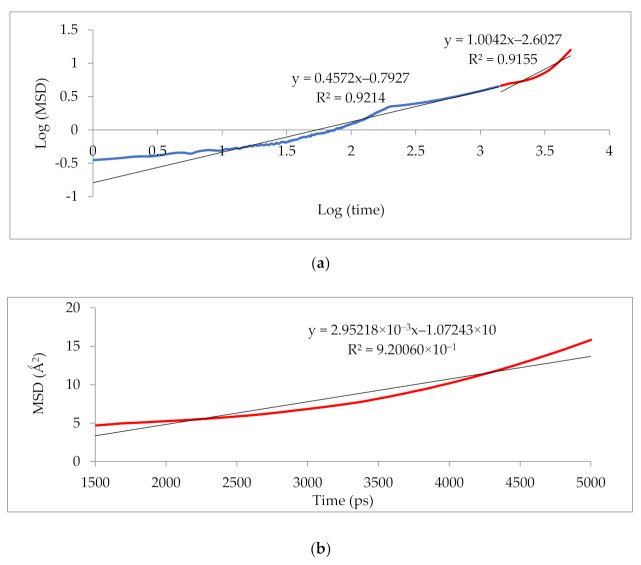
Mean square displacement (MSD) graph of (**a**) logarithmic plot for determination of Einstein diffusion region, and (**b**) Normal plot for evaluation of diffusivity coefficients.

Figure 10 illustrates that the simulation was run for 5000 ps to determine the anomalous (blue) from the Einstein (red) diffusion region for 25 wt.% silica at 30% CO_2_ gas concentration. Subsequently, the slope of the Einstein normal diffusion regime was used to determine the diffusivity.

**Figure 11:**
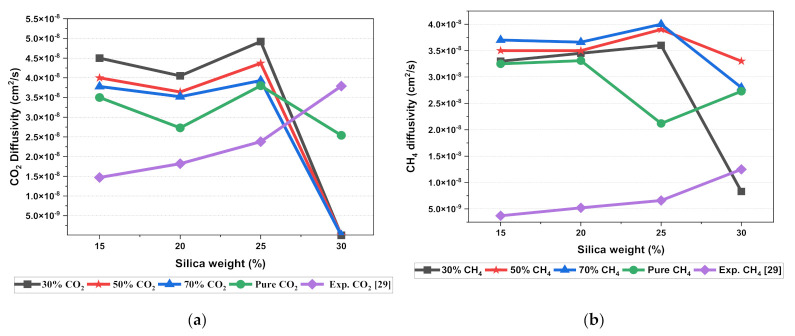

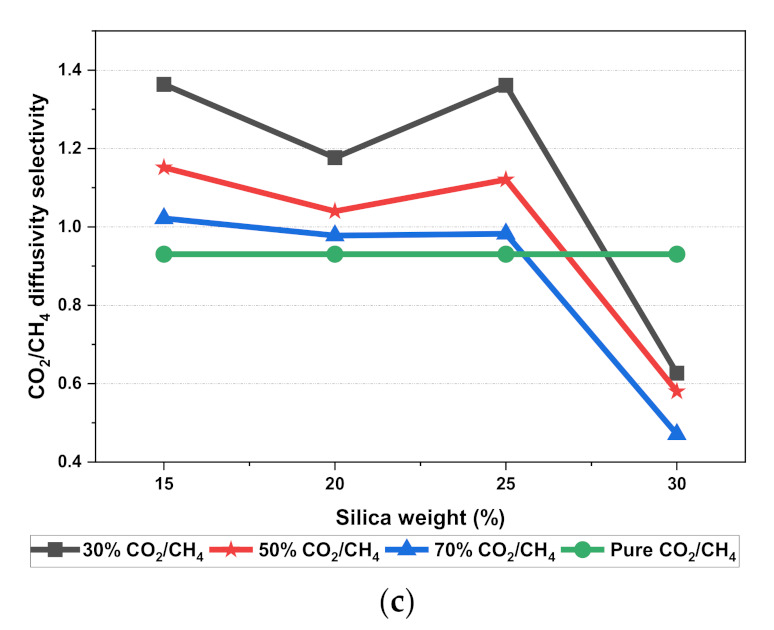
Diffusivity graph of silica/PSF-based MMM with varying mixed gas concentrations and filler weight percentage for (**a**) CO_2_ diffusivity, (**b**) CH_4_ diffusivity, and (**c**) CO_2_/CH_4_ selectivity.

The results illustrated that the diffusivity coefficients of the silica/PSF MMM increased with the weight percentage up to 25 wt.% followed by a decrease at 30 wt.% because silica nanocomposite particles disrupt the polymer chain by creating voids in the matrix. This diffusivity change was also perceived due to the higher diffusivity of CO_2_ than CH_4_ gas molecules [59,68,69]. However, in mixed gas conditions with an increasing weight percentage of the silica nanocomposites, the gas with the highest and faster diffusing ability suppresses the pathway of the other mixed gas molecules passing through the matrix. The same pattern was observed in this case for mixed gases when passing through the membrane matrix, CO_2_, which diffused faster than CH_4_ with a higher diffusivity value. The diffusivity increased with the increase in the weight percentage of the silica. The diffusivity values for the pure gas were found to be in close agreement with the values reported by Ahn et al.’s experimental study on silica/PSF MMM [29]. When compared with the pure gas condition passing through silica/PSF MMM, the diffusivity selectivity for mixed gases was less than that of the pure ones, as observed in Figure 11. Since the active energies among the single gas are more prominent than a mixed gas, the diffusivity selectivity was found to decrease with an increasing weight percentage of silica for mixed gas conditions [27].

#### 3.2.3. Gas Permeability (CO_2_/CH_4_)

**Figure 12:**
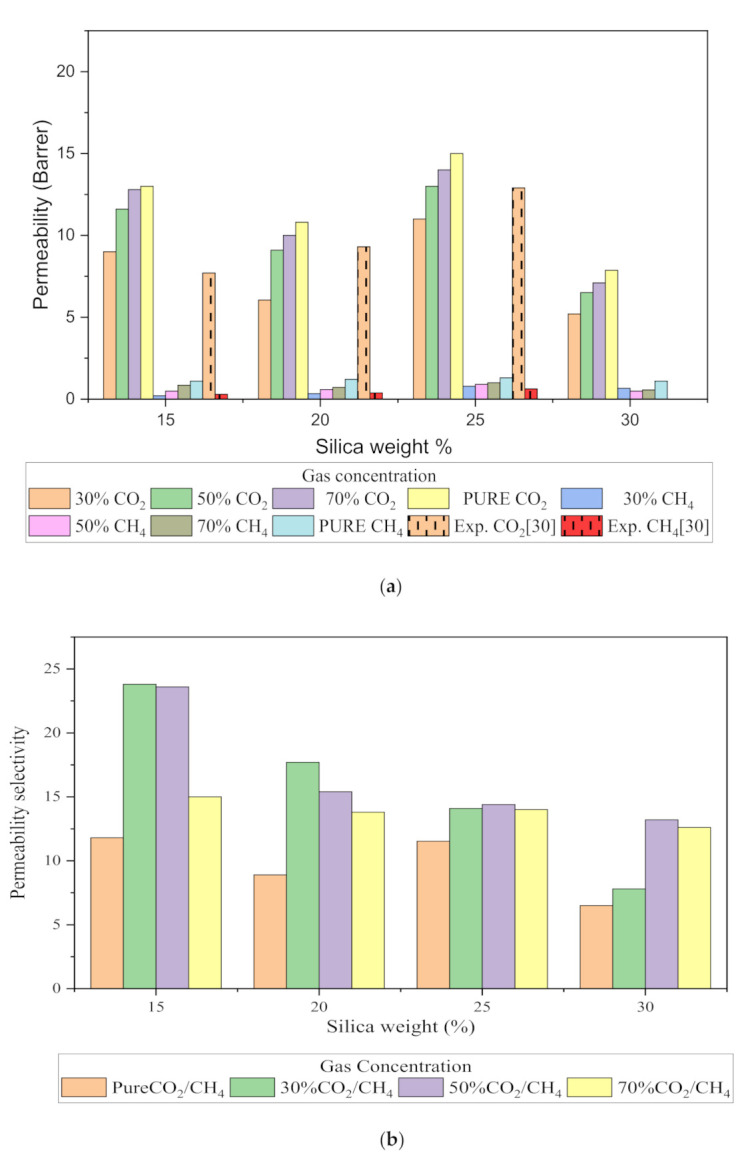
Permeability graph for (**a**) CO_2_ and CH_4_, and (**b**) CO_2_/CH_4_ selectivity with varying mixed gas concentrations and filler weight percentage.

The relative increase in diffusion coefficients increases the permeability of the gas molecules, and this is because of the increase in the packing percentage of the nanocomposites in the membrane matrix. As displayed in Figure 12, it was found that permeability was increased with the addition of silica due to increasing intermolecular spacing and free volume in the matrix. As permeation is also directly related to free volume, the simulation study showed that the free volume increased with the increase in the silica weight percentage up to 25 wt.%. However, the trend in the increase was found to be not consistent due to silica agglomeration for the 30 wt.% membrane structure. The enhancement in CH_4_ permeability was more pronounced with an increment in the void channel, which caused a slight compromise in CO_2_/CH_4_ selectivity. Hence, the permeability selectivity was observed to decrease with an increment in the silica weight percentage when more void spaces were available to promote transport of both gases. Similarly, for mixed gas conditions, a tradeoff between permeability and selectivity was observed. When compared to the pure gas transport in silica/PSF MMM, the increase in the permeation was observed to be more significant with a noticeable decrement in the selectivity of the mixed gas conditions due to the increase in the diffusivity for CO_2_. The overall permeability values were found to be smaller than the experimental values, which are limited to pure gas conditions [29]. From the results of diffusivity and solubility, it was observed that the diffusion coefficient was the dominating factor in governing the permeability of CO_2_ and CH_4_ in silica/PSF MMM as compared to the solubility coefficient.

### 3.3. Empirical Modeling Results

The parameters selected for the calculation of solubility and permeability of empirical modeling are listed in Table 5.

**Figure 13:**
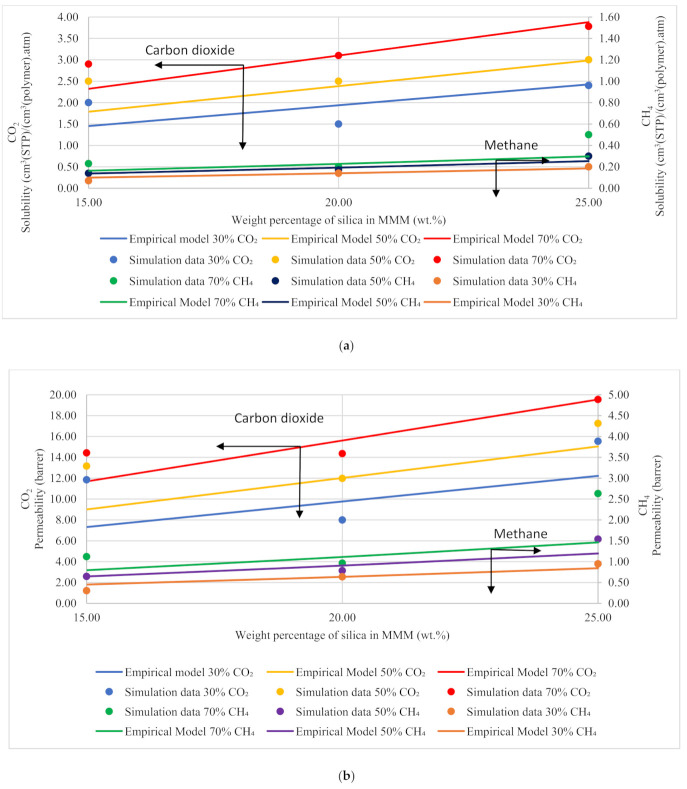
Development of an empirical model for silica/PSF MMMs with varying mixed gas concentrations and filler weight percentage for (**a**) solubility and (**b**) permeability.

Based on Equation (12), the gas molecules were assumed to be fully mobile in Henry’s environment, while being partially mobile in the Langmuir environment, which makes permeabilities of the *x* gas molecules concentration-independent in the filler ($P_{fi}$) and the polymer ($P_{ci}$) phases [51]. The data points in Figure 13 are the depiction of simulation data, whereas the trendline shows the empirical model calculations. The model results were compared to the simulation results, and the percentage error was overcome using GRG nonlinear solver iterations. The percentage error for solubility and permeability was found to be 18% and 15%, respectively, while the units for solubility and permeability were kept consistent with the simulation data. The model results depicted satisfactory results and showed a lesser difference for lower weight percentages of silica (i.e., 15 and 20 wt.%). The modeling results were also found to coincide with the simulation results with increasing gas concentrations for CH_4_ with a slight difference in CO_2_. Unlike the simulation results, the model results for 25 wt.% silica/PSF-based MMM displayed that the error was found to be increasing when compared with the simulation results. In the future, the model can contribute to overcoming the tradeoff in gas transport properties in the future with further proposed modifications and designs to obtain significant results for mixed gas conditions in real membrane separation.

The empirical model is demonstrated to be able to circumvent the contradictory prediction by the Maxwell model in earlier works while incorporating the effect of mixed gas concentrations towards gas transport behavior in PSF/silica MMM with varying filler loading [29]. This highlights the importance of molecular simulation work to select and to develop appropriate empirical models that can be used to describe gas transport behavior according to the filler–polymer system of interest in a relatively convenient and cost-efficient way as compared to experimental interrogation.

## 4. Conclusions

Currently, the MMM molecular simulations are confined to single gas conditions, which do not satisfy real separation system requirements. This research aimed to study the effect of silica nanoparticles on the transport behavior and properties of gases with varying concentrations in silica/PSF membranes. Molecular dynamic simulation techniques were carried out on the MMMs under mixed gas CO_2_/CH_4_ conditions and increasing silica weight percentages. A validated computational framework was presented with a compared analysis of simulation densities with published experimental results for the construction of the silica/PSF MMMs. Each simulated cell was subjected to analysis to study the free volume and the energy distribution at the equilibrium state. Concerning gas transport properties, the simulated results, in general, showed a consistent increase in the gas diffusivity, solubility, and permeability with a decrement in the selectivity for CO_2_/CH_4_ due to the increase in the free volume with increasing silica weight percentage. A consistent increase in gas transport properties was observed until agglomeration was encountered. The results for mixed gas solubility displayed a competitive gas transport in the membranes with increasing CO_2_ gas concentrations. The increase in the void space with increasing silica weight percentage enhanced the diffusion of the mixed gases. Consequently, the increase in the gas transport properties of the silica/polysulfone MMM for pure gas was higher with prominent active energies. However, it is notable that under pure and mixed gas conditions, the increase in the gas transport properties was observed up to 25 wt.% and decreased for 30 wt.% with increasing filler content. An empirical model was developed to quantify the effect of gas concentrations and weight percentage of inorganic filler on permeability and solubility. The model reveals convincing results when compared with the simulation results for both CO_2_ and CH_4_. It also highlights the advantage of the molecular simulation model to elucidate gas transport properties, which can be used to screen, evaluate, and develop an appropriate empirical model to describe the separation performance of membrane applied in macroscopic process simulation studies. Henceforth, it can be settled that the techniques used for molecular simulation and modeling in this research are reliable to achieve screening and design of new generation membranes by relating molecular information at the atomistic level to macroscopic membrane behavior and mixed gas transport properties while assisting in the development of empirical correlation for further quantification of its separation performance.

## Figures and Tables

**Table 1:** Modules, operating conditions, and parameters for molecular simulation work.

Modules	Specifications
Build/Geometric Optimization	Algorithm: Smart Quality: Medium Energy: 0.001 kcal/mol Force: 0.5 kcal/mol/A Max. iterations: 500 Energy: COMPASS
Amorphous Cell	Task: Construction Quality: Medium Temperature: 308.15 K Energy: COMPASS Initial Density: 1.20 g/cm^3^ (CO_2_/CH_4_)
FORCITE (Anneal)	Task: Anneal Quality: Medium Energy: COMPASS Annealing Cycles: 10 Initial Temperature: 308.15 K Final Temperature: 508.15 K Pressure: 1.013 × 10^−4^ kPa Time step: 1.0 fs
FORCITE(Dynamics)	Task: Dynamics Quality: Medium Energy: COMPASS Ensemble: Canonical (NVT) and Isothermal–Iso baric (NPT) Barostat: Andersen (0.5 ps cell time constant) Thermostat: NHL (0.005 Q ratio) Time: 500 ps
Sorption	Task: Adsorption Isotherm Method: Metropolis Quality: Medium Energy: COMPASS Pressure: 1.0 × 10^−4^ (kPa)–101.33 (kPa)
FORCITE Analysis (Scatter)	Radiation: X-ray Cutoff: 8.84 Å 2-Theta: 5.00° Wavelength: 1.54178 Å (10°–45°)

**Table 2:** The input of pristine materials used for simulating MMMs with different silica wt.%.

Silica Weight %	No. of Polysulfone Chains in Simulated MMM	No. of Silica Nanoparticles in Simulated MMM	Cell Length Å after Equilibration
15	1	1	23.97 Å
20	2	3	30.97 Å
25	1	2	25.13 Å
30	3	5	36.25 Å

**Table 3:** Density data for simulating MMMs with different silica wt.%.

Silica Weight Percentage	Simulated Density for Silica/PSF MMM (g/cm^3^)	Experimental Density (g/cm^3^) [29]	Percentage Error	Simulated Density for Silica/PSF MMM with the Inclusion of CO_2_, CH_4_ (g/cm^3^)
15	1.26	1.28	1.55%	1.26
20	1.25	1.31	4.58%	1.24
25	1.27	1.35	5.93%	1.26
30	1.31	1.39	5.75%	1.30

**Table 4:** XRD analysis for simulated silica/PSF MMMs with different silica wt.%.

Scheme 1	2θ	d-Spacing Å
15	17.2	5.12 (5.25) ^a^
20	16.8	5.28 (5.43) ^a^
25	16.5	5.36 (5.43) ^a^
30	16.7	5.30

^a^ Simulated d-spacing for silica/PSF MMM by Golzar et al., 2014 [19].

**Table 5:** Parameters for empirical modeling.

-	CO_2_	CH_4_
**Solubility**	-	-
**$K_{f}$**	2.86	13.86
**$C_{f}^{s}$**	39.65	3.21
**$C_{c}^{s}$**	5.22 × 10^−1^	25.41
**$K_{h}$**	0.20456	1.285
**$K_{c}$**	0.04477	15.376
**Permeability**	-	-
**$K_{f}$**	2.865	13.8612
**$C_{f}^{s}$**	39.659	3.210
**$C_{c}^{s}$**	5.22 × 10^−1^	25.41
**$K_{h}$**	0.204	1.285
**$K_{c}$**	0.044	15.37
**$D_{f}$**	5.050	6.446
**$D_{h}$**	1.130	3.91 × 10^−1^
**$D_{c}$**	1.023	1.493

## Data Availability

The data presented in this study are available upon request from the corresponding author.

## Abbreviations

*C_i_*	The concentration of gas species *i* in the polymer
*C_fi_*	The capacity of the gas species *i* in the filler
*C^s^_ci_*	The capacity of the gas species *i* in the polymer
*CO*_2_	Carbon dioxide
*CH*_4_	Methane
*K_fi_*	Affinity constant of gas species *i* in the filler
*f*	Fugacity of the gas species
*D_i_*	Diffusivity coefficient of gas species *i*
*D_fi_*	Diffusion of gas species *i* in the filler
*D_hi_*	Diffusivity in Henry’s sites in the polymer
*D_ci_*	Diffusivity in Langmuir sites in the polymer
*FFV*	Fractional free volume
*H*_2_	Hydrogen
*K_hi_*	Henry’s constant for gas species *i* in polymer
*K_ci_*	The affinity constant for gas species *i* in polymer
*N*	No. of molecules
*n*	Exponential parameter
*N*_2_	Nitrogen
*O*_2_	Oxygen
*P*	Pressure (atm)
*P(E)*	The potential energy of configuration (kcal/mol)
*P_i_*	Permeability of the gas species i
*Q*	Magnitude scattering vector
*r_i_*	The position vector of an atom i for the mass center of the molecule (Å)
*S_i_*	Solubility coefficient of gas species *i*
*S_fi_*	Solubility of gas species *i* in the filler
*STP*	Standard temperature and pressure
*T*	Temperature (K)
*T_g_*	Glass transition temperature
*V_o_*	The occupied volume of the polymer chains (Å^3^)
*V_s_*	The specific volume of the polymer chains (Å^3^)
*wt.%*	Weight percent
*x_i_*	Mole fraction of gas species *i*
*φ*	The weight percentage of the filler (silica)
λ	Radiation wavelength
2θ	Scattering angle
